# (*E*)-4-Hy­droxy-*N*′-(2-hy­droxy-5-iodo­benzyl­idene)benzohydrazide methanol monosolvate

**DOI:** 10.1107/S1600536812034848

**Published:** 2012-08-11

**Authors:** Parisa Mahboubi Anarjan, Hassan Hosseini Monfared, N. Burcu Arslan, Canan Kazak, Rahman Bikas

**Affiliations:** a Sama Technical and Vocational Training College, Islamic Azad University, Mamaghan Branch, Mamaghan, Iran; b Department of Chemistry, Faculty of Science, University of Zanjan, 45195-313 Zanjan, Iran; c Department of Physics, Faculty of Arts and Sciences, Ondokuz Mayis University, 55019 Kurupelit, Samsun, Turkey

## Abstract

In the title compound, C_14_H_11_IN_2_O_3_·CH_4_O, the dihedral angle between the benzene rings is 33.2 (3)°. The mol­ecule displays *trans* and *anti* conformations about the C=N and N—N bonds, respectively. There is an intra­molecular O—H⋯N(azomethine) hydrogen bond. Inter­molecular N—H⋯O and O—H⋯O hydrogen bonds consolidate mol­ecules into a three-dimensional architecture.

## Related literature

For the structures of related carbohydrazides, see: Monfared *et al.* (2010*a* ▶); Bikas *et al.* (2010*a* ▶,*b* ▶, 2012*a* ▶,*b* ▶). For catalytic applications of aroylhydrazones, see: Monfared *et al.* (2010*b* ▶).
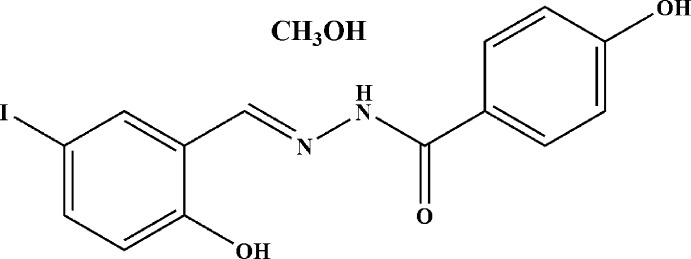


## Experimental

### 

#### Crystal data

C_14_H_11_IN_2_O_3_·CH_4_O
*M* *_r_* = 414.19
Monoclinic, 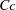

*a* = 10.1077 (7) Å
*b* = 12.5703 (11) Å
*c* = 13.1586 (17) Å
β = 102.886 (10)°
*V* = 1629.8 (3) Å^3^
*Z* = 4
Mo *K*α radiation
μ = 1.98 mm^−1^
*T* = 293 K
0.3 × 0.3 × 0.3 mm

#### Data collection

Agilent SuperNova (Single source at offset), Eos diffractometer
Absorption correction: multi-scan (*CrysAlis PRO*; Agilent, 2012 ▶) *T*_min_ = 0.864, *T*_max_ = 1.000
3370 measured reflections
2094 independent reflections
1981 reflections with *I* > 2σ(*I*)
*R*_int_ = 0.034

#### Refinement

*R*[*F* ^2^ > 2σ(*F* ^2^)] = 0.036
*wR*(*F* ^2^) = 0.090
*S* = 1.04
2094 reflections
201 parameters
2 restraints
H-atom parameters constrained
Δρ_max_ = 0.32 e Å^−3^
Δρ_min_ = −0.56 e Å^−3^
Absolute structure: Flack (1983 ▶), 419 Friedel pairs
Flack parameter: −0.01 (3)

### 

Data collection: *CrysAlis PRO* (Agilent, 2012 ▶); cell refinement: *CrysAlis PRO*; data reduction: *CrysAlis PRO*; program(s) used to solve structure: *SHELXS97* (Sheldrick, 2008 ▶); program(s) used to refine structure: *SHELXL97* (Sheldrick, 2008 ▶); molecular graphics: *DIAMOND* (Brandenburg, 2006 ▶); software used to prepare material for publication: *WinGX* (Farrugia, 1999 ▶) and *PLATON* (Spek, 2009 ▶).

## Supplementary Material

Crystal structure: contains datablock(s) I, global. DOI: 10.1107/S1600536812034848/tk5140sup1.cif

Structure factors: contains datablock(s) I. DOI: 10.1107/S1600536812034848/tk5140Isup2.hkl

Supplementary material file. DOI: 10.1107/S1600536812034848/tk5140Isup3.cml

Additional supplementary materials:  crystallographic information; 3D view; checkCIF report

## Figures and Tables

**Table 1:** Hydrogen-bond geometry (Å, °)

*D*—H⋯*A*	*D*—H	H⋯*A*	*D*⋯*A*	*D*—H⋯*A*
O1—H1⋯N1	0.82	1.89	2.607 (8)	146
N2—H2⋯O4	0.86	2.06	2.897 (7)	164
O3—H3⋯O2^i^	0.82	1.90	2.712 (6)	171
O4—H4⋯O2^ii^	0.82	2.05	2.868 (7)	177

Symmetry codes: (i) 
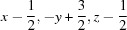
; (ii) 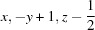.

**Fig. 1.**
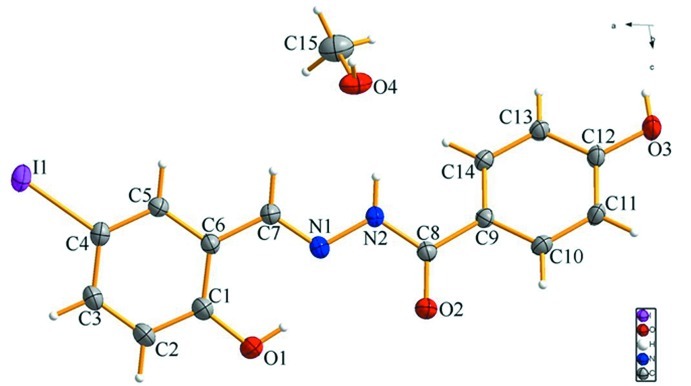
The molecular structure of the title compound. Displacement ellipsoids are drawn at the 30% probability level.

**Fig. 2.**
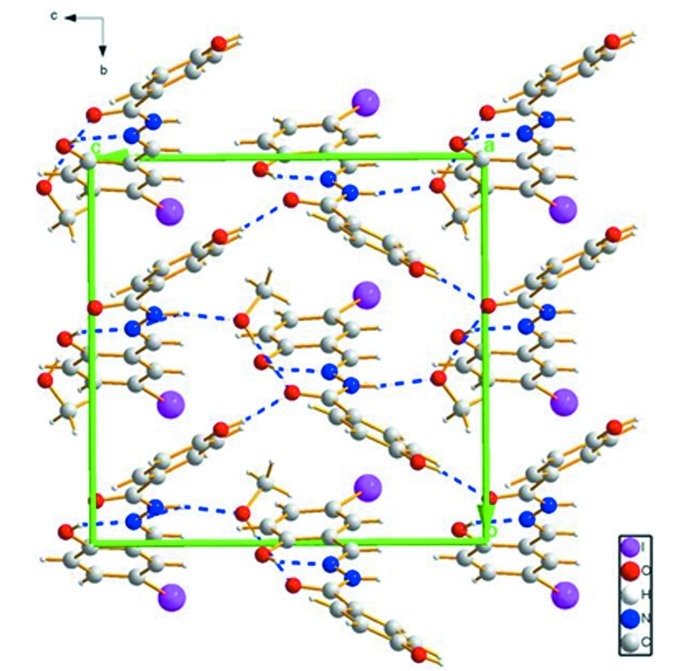
A packing diagram of the title compound. The blue dashed lines indicate intra- and inter-molecular hydrogen bonds.

## supplementary crystallographic information

### Comment

Hydrazones are a special group of compounds in the Schiff base family that are characterized by the presence of *RR*'C=N–N=C(O)*R*'' which two inter-linked nitrogen atoms (–N—N–) separate them into a different class from imines, oximes, *etc*. Hydrazone ligands derived from the condensation of acid hydrazides (R–CO–NH–NH_2_) with aromatic carbonyl compounds are important O, N-donor ligands. Hydrazone derivatives have widespread applications in fields such as coordination chemistry, bioinorganic chemistry, magnetics, electronics, nonlinear optics and fluorescent materials. Aroylhydrazone complexes also seem to be good candidates for catalytic oxidation studies because of their resistance to oxidation (Monfared *et al.*, 2010*b*).

As part of our studies on the synthesis and characterization of hydrazone derivatives (Bikas *et al.*, 2010*a*,*b*; Bikas *et al.*, 2012*a*,*b*), we report here the crystal structure of [(*E*)-4-hydroxy-*N*'-(2-hydroxy-5-iodobenzylidene)benzohydrazide] methanol solvate. The asymmetric unit of C_14_H_11_IN_2_O_3_.CH_4_O contains one molecule of hydrazone and a molecule of methanol, as shown in Fig. 1. In the title compound, the bond distances are in the normal range for similar hydrazone compounds (Monfared *et al.*, 2010*a*; Bikas *et al.*, 2012*a*,*b*). The dihedral angle between the mean planes of the phenol ring and the salcylidine ring is 33.2 (3)°. Molecule adopts an *E* configuration with respect to the C7=N1 bond. There is an intramolecular O—H···N hydrogen bond between the hydroxyl group and imine nitrogen atom, Table 1. In the crystal structure, the O atom of methanol molecule accepts a hydrogen bond from an amine H atom (NH), and forms another intermolecular *O*—*H*···*O*(carbonyl) hydrogen bond, thereby linking two carbohydrazide molecules. The result is a supramolecular layer parallel to (010). The carbonyl O atom accepts another O—H···O hydrogen bond which the O—H phenol is a donor group (Table 1, Fig. 2).

### Experimental

For preparing the title compound, a methanol (10 ml) solution of 2-hydroxy-5-iodobenzaldehyde (1.5 mmol) was added drop-wise to a methanol solution (10 ml) of 4-hydroxybenzoic acid hydrazide (1.5 mmol). The mixture was refluxed for 5 h. The solution was evaporated on a steam-bath to 5 ml and cooled to room temperature. White precipitates of the title compound were separated and filtered off, washed with 3 ml of cooled methanol and then dried in air. Colourless crystals of the title compound were obtained from its methanol solution by slow solvent evaporation. Yield 94%. Selected IR (cm^-1^): 3446 (*s*, broad, O—H), 3224 (*s*, N—H), 1626 (*vs*), 1577 (*m*), 1509 (*s*), 1278 (*vs*), 1013 (*s*), 850 (*m*), 690 (*m*).

### Refinement

The hydrogen atoms of the N—H and O—H groups were positioned geometrically and refined as riding atoms with, N—H = 0.86 Å and *U*(H) = 1.2*U*_eq_(N), and with O—H = 0.82 Å and *U*(H) = 1.5*U*_eq_(O). The C—H hydrogen atoms were positioned geometrically and refined as riding atoms with C—H = 0.93 Å and *U*(H) = 1.2*U*_eq_(C) for aromatic-hydrogen atoms, and C—H = 0.96 Å and *U*(H) = 1.2 *U*_eq_(C) for the methyl group.

### Crystal data

**Table:**

C_14_H_11_IN_2_O_3_·CH_4_O	*F*(000) = 816
*M**_r_* = 414.19	*D*_x_ = 1.688 Mg m^−^^3^
Monoclinic, *C**c*	Mo *K*α radiation, λ = 0.71073 Å
Hall symbol: C -2yc	Cell parameters from 1140 reflections
*a* = 10.1077 (7) Å	θ = 3.3–32.3°
*b* = 12.5703 (11) Å	µ = 1.98 mm^−^^1^
*c* = 13.1586 (17) Å	*T* = 293 K
β = 102.886 (10)°	Block, colourless
*V* = 1629.8 (3) Å^3^	0.3 × 0.3 × 0.3 mm
*Z* = 4	

### Data collection

**Table:**

Agilent SuperNova (Single source at offset), Eos diffractometer	2094 independent reflections
Radiation source: SuperNova (Mo) X-ray Source	1981 reflections with *I* > 2σ(*I*)
Mirror monochromator	*R*_int_ = 0.034
Detector resolution: 16.0454 pixels mm^-1^	θ_max_ = 26.4°, θ_min_ = 3.3°
ω scans	*h* = −12→8
Absorption correction: multi-scan (*CrysAlis PRO*; Agilent, 2012)	*k* = −13→15
*T*_min_ = 0.864, *T*_max_ = 1.000	*l* = −16→14
3370 measured reflections	

### Refinement

**Table:**

Refinement on *F*^2^	Secondary atom site location: difference Fourier map
Least-squares matrix: full	Hydrogen site location: inferred from neighbouring sites
*R*[*F*^2^ > 2σ(*F*^2^)] = 0.036	H-atom parameters constrained
*wR*(*F*^2^) = 0.090	*w* = 1/[σ^2^(*F*_o_^2^) + (0.0466*P*)^2^] where *P* = (*F*_o_^2^ + 2*F*_c_^2^)/3
*S* = 1.04	(Δ/σ)_max_ < 0.001
2094 reflections	Δρ_max_ = 0.32 e Å^−^^3^
201 parameters	Δρ_min_ = −0.56 e Å^−^^3^
2 restraints	Absolute structure: Flack (1983), 419 Friedel pairs
Primary atom site location: structure-invariant direct methods	Flack parameter: −0.01 (3)

### Special details

**Table:**

Geometry. All e.s.d.'s (except the e.s.d. in the dihedral angle between two l.s. planes) are estimated using the full covariance matrix. The cell e.s.d.'s are taken into account individually in the estimation of e.s.d.'s in distances, angles and torsion angles; correlations between e.s.d.'s in cell parameters are only used when they are defined by crystal symmetry. An approximate (isotropic) treatment of cell e.s.d.'s is used for estimating e.s.d.'s involving l.s. planes.
Refinement. Refinement of *F*^2^ against ALL reflections. The weighted *R*-factor *wR* and goodness of fit *S* are based on *F*^2^, conventional *R*-factors *R* are based on *F*, with *F* set to zero for negative *F*^2^. The threshold expression of *F*^2^ > σ(*F*^2^) is used only for calculating *R*-factors(gt) *etc*. and is not relevant to the choice of reflections for refinement. *R*-factors based on *F*^2^ are statistically about twice as large as those based on *F*, and *R*- factors based on ALL data will be even larger.

### Fractional atomic coordinates and isotropic or equivalent isotropic displacement parameters (Å^2^)

**Table:**

	*x*	*y*	*z*	*U*_iso_*/*U*_eq_	
I1	0.71478 (4)	0.35952 (3)	0.30013 (4)	0.05205 (16)	
O1	0.3254 (6)	0.5311 (5)	0.5685 (4)	0.0672 (17)	
H1	0.2521	0.5492	0.5321	0.101*	
O2	−0.0512 (5)	0.6112 (4)	0.4916 (3)	0.0450 (11)	
O3	−0.5681 (5)	0.7936 (4)	0.1732 (3)	0.0548 (13)	
H3	−0.5634	0.8156	0.1155	0.082*	
O4	0.0029 (7)	0.5759 (4)	0.1189 (4)	0.0613 (15)	
H4	−0.0129	0.5213	0.0842	0.092*	
N1	0.1430 (6)	0.5574 (5)	0.3960 (4)	0.0412 (12)	
N2	0.0194 (5)	0.5934 (4)	0.3409 (4)	0.0379 (11)	
H2	0.0008	0.5958	0.2739	0.045*	
C1	0.4061 (7)	0.4922 (6)	0.5066 (5)	0.0469 (16)	
C2	0.5354 (8)	0.4584 (7)	0.5544 (5)	0.0553 (19)	
H2A	0.5635	0.4611	0.6266	0.066*	
C3	0.6226 (7)	0.4209 (6)	0.4960 (5)	0.0472 (16)	
H3A	0.7102	0.4002	0.5285	0.057*	
C4	0.5793 (6)	0.4142 (5)	0.3886 (4)	0.0376 (13)	
C5	0.4500 (6)	0.4478 (5)	0.3393 (4)	0.0369 (13)	
H5	0.4225	0.4441	0.2671	0.044*	
C6	0.3614 (6)	0.4868 (5)	0.3973 (4)	0.0359 (13)	
C7	0.2277 (6)	0.5220 (5)	0.3436 (5)	0.0395 (14)	
H7	0.2027	0.5190	0.2712	0.047*	
C8	−0.0721 (7)	0.6251 (4)	0.3958 (4)	0.0336 (13)	
C9	−0.1990 (6)	0.6748 (5)	0.3356 (4)	0.0318 (12)	
C10	−0.3134 (7)	0.6734 (6)	0.3778 (5)	0.0407 (14)	
H10	−0.3078	0.6452	0.4439	0.049*	
C11	−0.4344 (7)	0.7135 (6)	0.3224 (5)	0.0426 (15)	
H11	−0.5112	0.7094	0.3502	0.051*	
C12	−0.4438 (6)	0.7604 (5)	0.2252 (4)	0.0371 (13)	
C13	−0.3289 (6)	0.7659 (5)	0.1848 (4)	0.0374 (13)	
H13	−0.3338	0.7982	0.1204	0.045*	
C14	−0.2068 (7)	0.7242 (5)	0.2388 (5)	0.0369 (14)	
H14	−0.1301	0.7288	0.2110	0.044*	
C15	0.0531 (14)	0.6548 (7)	0.0605 (7)	0.075 (3)	
H15A	−0.0125	0.7109	0.0429	0.112*	
H15B	0.0696	0.6238	−0.0022	0.112*	
H15C	0.1363	0.6833	0.1013	0.112*	

### Atomic displacement parameters (Å^2^)

**Table:**

	*U*^11^	*U*^22^	*U*^33^	*U*^12^	*U*^13^	*U*^23^
I1	0.0380 (2)	0.0592 (3)	0.0620 (3)	0.0113 (3)	0.01773 (16)	−0.0025 (2)
O1	0.054 (3)	0.108 (5)	0.039 (2)	0.030 (4)	0.010 (2)	−0.005 (2)
O2	0.043 (3)	0.057 (3)	0.035 (2)	0.009 (2)	0.0088 (19)	0.0027 (18)
O3	0.036 (3)	0.080 (4)	0.052 (3)	0.020 (3)	0.016 (2)	0.019 (2)
O4	0.083 (4)	0.062 (3)	0.040 (2)	−0.015 (3)	0.015 (2)	−0.007 (2)
N1	0.031 (3)	0.053 (3)	0.038 (2)	0.006 (3)	0.005 (2)	0.002 (2)
N2	0.030 (3)	0.048 (3)	0.035 (2)	0.009 (3)	0.006 (2)	−0.002 (2)
C1	0.041 (4)	0.060 (4)	0.040 (3)	0.011 (3)	0.007 (3)	0.004 (3)
C2	0.042 (4)	0.079 (5)	0.041 (3)	0.015 (4)	0.000 (3)	0.005 (3)
C3	0.032 (3)	0.059 (4)	0.048 (3)	0.008 (3)	0.003 (3)	0.004 (3)
C4	0.032 (3)	0.038 (3)	0.045 (3)	0.002 (3)	0.012 (3)	0.000 (2)
C5	0.034 (3)	0.043 (3)	0.032 (3)	0.003 (3)	0.005 (2)	−0.002 (2)
C6	0.030 (3)	0.039 (3)	0.036 (3)	0.003 (3)	0.004 (2)	−0.001 (2)
C7	0.032 (3)	0.053 (4)	0.034 (3)	0.002 (3)	0.007 (2)	−0.001 (2)
C8	0.031 (3)	0.034 (3)	0.035 (3)	0.001 (2)	0.006 (2)	−0.001 (2)
C9	0.032 (3)	0.031 (3)	0.032 (3)	0.005 (2)	0.007 (2)	−0.003 (2)
C10	0.040 (4)	0.051 (4)	0.035 (3)	0.004 (3)	0.016 (3)	0.005 (3)
C11	0.035 (3)	0.057 (4)	0.041 (3)	0.012 (3)	0.019 (3)	0.010 (3)
C12	0.030 (3)	0.041 (3)	0.043 (3)	0.009 (3)	0.012 (2)	0.004 (2)
C13	0.034 (3)	0.044 (3)	0.036 (3)	0.004 (3)	0.012 (2)	0.009 (2)
C14	0.032 (3)	0.047 (4)	0.034 (3)	−0.001 (3)	0.012 (3)	0.001 (2)
C15	0.099 (9)	0.066 (5)	0.061 (5)	−0.003 (5)	0.021 (5)	−0.003 (4)

### Geometric parameters (Å, º)

**Table:**

I1—C4	2.101 (6)	C5—C6	1.389 (9)
O1—C1	1.365 (9)	C5—H5	0.9300
O1—H1	0.8200	C6—C7	1.447 (8)
O2—C8	1.243 (7)	C7—H7	0.9300
O3—C12	1.355 (7)	C8—C9	1.486 (8)
O3—H3	0.8200	C9—C10	1.390 (9)
O4—C15	1.416 (12)	C9—C14	1.403 (8)
O4—H4	0.8200	C10—C11	1.373 (9)
N1—C7	1.292 (9)	C10—H10	0.9300
N1—N2	1.374 (7)	C11—C12	1.392 (9)
N2—C8	1.355 (8)	C11—H11	0.9300
N2—H2	0.8600	C12—C13	1.383 (9)
C1—C2	1.385 (10)	C13—C14	1.383 (9)
C1—C6	1.409 (8)	C13—H13	0.9300
C2—C3	1.375 (10)	C14—H14	0.9300
C2—H2A	0.9300	C15—H15A	0.9600
C3—C4	1.387 (9)	C15—H15B	0.9600
C3—H3A	0.9300	C15—H15C	0.9600
C4—C5	1.388 (8)		
			
C1—O1—H1	109.5	O2—C8—N2	121.2 (6)
C12—O3—H3	109.5	O2—C8—C9	122.1 (6)
C15—O4—H4	109.5	N2—C8—C9	116.7 (5)
C7—N1—N2	117.7 (5)	C10—C9—C14	119.0 (6)
C8—N2—N1	117.7 (5)	C10—C9—C8	118.5 (5)
C8—N2—H2	121.2	C14—C9—C8	122.5 (6)
N1—N2—H2	121.2	C11—C10—C9	120.4 (6)
O1—C1—C2	117.9 (6)	C11—C10—H10	119.8
O1—C1—C6	121.9 (6)	C9—C10—H10	119.8
C2—C1—C6	120.3 (7)	C10—C11—C12	120.9 (6)
C3—C2—C1	120.6 (6)	C10—C11—H11	119.6
C3—C2—H2A	119.7	C12—C11—H11	119.6
C1—C2—H2A	119.7	O3—C12—C13	123.6 (5)
C2—C3—C4	119.6 (6)	O3—C12—C11	117.3 (6)
C2—C3—H3A	120.2	C13—C12—C11	118.9 (6)
C4—C3—H3A	120.2	C12—C13—C14	120.9 (5)
C3—C4—C5	120.5 (6)	C12—C13—H13	119.6
C3—C4—I1	119.2 (5)	C14—C13—H13	119.6
C5—C4—I1	120.2 (4)	C13—C14—C9	119.9 (6)
C4—C5—C6	120.4 (5)	C13—C14—H14	120.1
C4—C5—H5	119.8	C9—C14—H14	120.1
C6—C5—H5	119.8	O4—C15—H15A	109.5
C5—C6—C1	118.6 (5)	O4—C15—H15B	109.5
C5—C6—C7	119.0 (5)	H15A—C15—H15B	109.5
C1—C6—C7	122.4 (6)	O4—C15—H15C	109.5
N1—C7—C6	120.1 (5)	H15A—C15—H15C	109.5
N1—C7—H7	119.9	H15B—C15—H15C	109.5
C6—C7—H7	119.9		
			
C7—N1—N2—C8	176.5 (6)	N1—N2—C8—O2	−7.8 (9)
O1—C1—C2—C3	−178.4 (7)	N1—N2—C8—C9	173.5 (5)
C6—C1—C2—C3	1.1 (12)	O2—C8—C9—C10	−21.6 (9)
C1—C2—C3—C4	−1.8 (12)	N2—C8—C9—C10	157.1 (6)
C2—C3—C4—C5	1.8 (10)	O2—C8—C9—C14	157.4 (6)
C2—C3—C4—I1	178.9 (6)	N2—C8—C9—C14	−23.9 (8)
C3—C4—C5—C6	−1.2 (10)	C14—C9—C10—C11	4.3 (10)
I1—C4—C5—C6	−178.2 (5)	C8—C9—C10—C11	−176.6 (6)
C4—C5—C6—C1	0.5 (10)	C9—C10—C11—C12	−2.8 (11)
C4—C5—C6—C7	179.5 (6)	C10—C11—C12—O3	177.0 (7)
O1—C1—C6—C5	179.0 (7)	C10—C11—C12—C13	0.1 (10)
C2—C1—C6—C5	−0.4 (11)	O3—C12—C13—C14	−175.6 (6)
O1—C1—C6—C7	0.0 (11)	C11—C12—C13—C14	1.1 (10)
C2—C1—C6—C7	−179.5 (7)	C12—C13—C14—C9	0.4 (10)
N2—N1—C7—C6	177.6 (6)	C10—C9—C14—C13	−3.1 (10)
C5—C6—C7—N1	179.2 (6)	C8—C9—C14—C13	177.9 (6)
C1—C6—C7—N1	−1.8 (10)		

### Hydrogen-bond geometry (Å, º)

**Table:**

*D*—H···*A*	*D*—H	H···*A*	*D*···*A*	*D*—H···*A*
O1—H1···N1	0.82	1.89	2.607 (8)	146
N2—H2···O4	0.86	2.06	2.897 (7)	164
O3—H3···O2^i^	0.82	1.90	2.712 (6)	171
O4—H4···O2^ii^	0.82	2.05	2.868 (7)	177

Symmetry codes: (i) *x*−1/2, −*y*+3/2, *z*−1/2; (ii) *x*, −*y*+1, *z*−1/2.
